# Effective elements of school health promotion across behavioral domains: a systematic review of reviews

**DOI:** 10.1186/1471-2458-9-182

**Published:** 2009-06-12

**Authors:** Louk WH Peters, Gerjo Kok, Geert TM Ten Dam, Goof J Buijs, Theo GWM Paulussen

**Affiliations:** 1 Graduate School of Teaching and Learning, University of Amsterdam, Amsterdam, The Netherlands; 2 Department of Prevention and Health Care, TNO (Netherlands Organisation for Applied Scientific Research) Quality of Life, Leiden, The Netherlands; 3 Faculty of Psychology, Maastricht University, Maastricht, The Netherlands; 4 Netherlands Institute for Health Promotion NIGZ, Woerden, The Netherlands

## Abstract

**Background:**

Most school health education programs focus on a single behavioral domain. Integrative programs that address multiple behaviors may be more efficient, but only if the elements of change are similar for these behaviors. The objective of this study was to examine which effective elements of school health education are similar across three particular behavioral domains.

**Methods:**

A systematic review of reviews of the effectiveness of school-based health promotion programs was conducted for the domains of substance abuse, sexual behavior, and nutrition. The literature search spanned the time period between 1995 and October 2006 and included three databases, websites of review centers and backward search. Fifty-five reviews and meta-analyses met predetermined relevance and publication criteria and were included. Data was extracted by one reviewer and checked by a second reviewer. A standardized data extraction form was used, with detailed attention to effective elements pertaining to program goals, development, content, methods, facilitator, components and intensity. Two assessors rated the quality of reviews as strong, moderate or weak. We included only strong and moderate reviews in two types of analysis: one based on interpretation of conflicting results, the other on a specific vote-counting rule.

**Results:**

Thirty six reviews were rated strong, 6 moderate, and 13 weak. A multitude of effective elements was identified in the included reviews and many elements were similar for two or more domains. In both types of analysis, five elements with evidence from strong reviews were found to be similar for all three domains: use of theory; addressing social influences, especially social norms; addressing cognitive-behavioral skills; training of facilitators; and multiple components. Two additional elements had positive results in all domains with the rule-based method of analysis, but had inconclusive results in at least one domain with the interpretion-based method of analysis: parent involvement and a larger number of sessions.

**Conclusion:**

Five effective elements of school health promotion were found to be similar across the three behavioral domains examined (substance abuse, sexual behavior, nutrition). An integrative program that addresses the three domains seems feasible. The five elements are primary candidates to include in programs targeting these behaviors.

## Background

Adolescents are a popular target group for health education and promotion programs because many health-risk behaviors, which contribute to the leading causes of morbidity and mortality among youth and adults, develop or augment during adolescence [1,2]. These behaviors include use of tobacco, alcohol and other substances, unprotected sexual activity, poor dietary habits, physical inactivity, and behaviors that contribute to unintentional injuries and violence. More and more evidence shows that several of these behaviors tend to co-occur [3-9] and have similar determinants [8,10], which opens up opportunities for integrative programs that address multiple behaviors [11]. Yet, most adolescent health promotion programs continue to address only one behavioral domain.

The majority of adolescent health promotion programs are intended for use in schools, often as a supplement to the regular curriculum. In many countries school staff feel overwhelmed by the ever-increasing supply of prevention programs, especially since they are faced with overcrowded curricula and limited opportunities for implementing prevention programs [12,13]. Integrative programs that address multiple risk behaviors effectively and efficiently may reduce the burden on schools and teachers [14]. Several authors have suggested that integrative programs can be efficient if the change processes or effective elements for different health behaviors are similar [6,15].

The observation that most programs focus on a single behavior also holds for the review literature that discusses effectiveness and effective elements of school-based health promotion. As Prochaska [[11], p. 283] argues, "science tends to value specificity, and specialists are trained to know what is specific to their disciplines rather than what is common across disciplines". Although many authors have observed that elements of effective programs appear to be similar across different behaviors [16-19], only a few authors have yet examined these commonalities systematically [16]. Knowledge of the similarities and dissimilarities of effective programs across behavioral domains may not only contribute to the development or elaboration of integrative programs. It may also deepen our understanding of what does and does not work in school health promotion and may contribute to transfer of knowledge and ideas from one domain to another.

The present review focuses on similarities between effective elements of school health education programs across three behavioral domains: substance abuse, sexual behavior and healthy nutrition. It was conducted to inform development of an integrative educational program that addresses all three domains. These domains were selected because they are among the ones most frequently addressed in Dutch secondary schools [20].

In light of the task of assessing three domains and the extensive body of literature on effectiveness that already exists in these domains, we opted for a review-of-reviews approach. As Nation and colleagues [16] stated, prevention now has a sufficient knowledge base to begin a meta-assessment of the characteristics of effective prevention programming. More and more, reviews draw on previous reviews for making statements about effectiveness [e.g., [16,19,21-24]].

## Methods

### Literature searches and inclusion/exclusion criteria

Three internet databases (Pubmed, PsycINFO, ERIC) were searched for relevant reviews published between January 1995 and October 2006 by combining groups of keywords pertaining to school health promotion, effectiveness and the three health behavior domains (see Table 1), generating over 1600 papers. The number and types of databases searched can be considered comprehensive [19] and efficient for locating literature about effectiveness of health promotion [25]. Also, the internet sites of six international review initiatives were searched for relevant reviews (see Table 1) and reference lists of already retrieved publications were scanned for additional reviews.

**Table 1:** Databases and keywords used in search strategies

**Databases**			
**Pubmed keywords**	**PsycINFO keywords**	**ERIC keywords**	**Review initiatives websites**

**School health promotion:** Curriculum Health-education Health-promotion School-health-services Health-plan-implementation **Effectiveness:** Program-evaluation Evaluation-studies Risk-reduction-behavior **Behavior focus:** Smoking Alcohol-drinking Sex-education Diet Food-habits	**School health promotion:** Curriculum Curriculum-development Educational-programs Schools School-environment Health-education Health-promotion **Effectiveness:** Effectiveness Educational-program-evaluation Treatment-effectiveness-evaluation Health-attitudes Health-behavior Health-knowledge **Behavior focus:** Tobacco-smoking Alcohol-abuse Safe-sex Sex-education Sexuality Sexually-transmitted-diseases Food-intake Nutrition Health-behavior Lifestyle	**School health promotion:** Curriculum School-health-services Health-programs Health-education Comprehensive-school-health-education Intervention Instruction **Effectiveness:** Program-effectiveness Program-evaluation Program-implementation Outcomes-of-education Knowledge-level Feedback Learning **Behavior focus:** Tobacco Smoking Alcohol-education Drinking Substance-abuse Sex-education Sexuality Nutrition Nutrition-instruction Eating-habits	Campbell Collaboration Centre for Reviews and Dissemination, York UK Cochrane Collaboration Effective Public Health Practice Project, Hamilton Canada EPPI-Centre, London UK Guide to Community Preventive Services

Publication year: January 1995 – October 2006. Language: English.

Note: The keywords within one group of keywords (e.g., school health promotion) were combined with 'OR', the groups were combined with 'AND'.

Titles and abstracts of publications were screened for relevance, and in case of doubt, entire publications were checked. Reviews were deemed relevant if they: a) included a review of primary effect studies (reviews of reviews were excluded); b) focused on one or more of the targeted risk behaviors (substance abuse, early or unprotected sexual behavior, dietary behavior); c) focused on regular, secondary-school-age youth or adolescents (12–18 years); d) included school-based programs with an educational approach; and e) discussed programs implemented in western countries. Furthermore, reviews had to be written in English, be published in a peer-reviewed journal listed on the Thomson Scientific master journal list or by an international review initiative, and be available over the Internet or from university libraries in The Netherlands.

Fifty five reviews met these criteria and were included: 5 about multiple domains of our interest (mostly about substance abuse and sexual behavior) [26-30], 24 about substance abuse [31-54], 17 about sexual behavior [55-71] and 9 about nutrition [72-80].

Of the substance abuse reviews, 5 focused specifically on tobacco, 4 on alcohol and the remaining 15 addressed tobacco and/or alcohol, possibly in combination with other substances. All reviews about multiple domains addressed substance abuse and sexuality programs and two also included nutrition programs. As some of these reviews focused on specific types of programs (e.g. peer programs) and not so much on specific behavioral domains, the results were usually not discussed for each specific domain. Some reviews in the multiple behavior and nutrition categories also addressed behaviors outside our focus (e.g. exercise), but results for these additional behaviors were not recorded.

### Data extraction

A standardized form (available from the first author) containing 27 categories was used for recording information about the characteristics and results of the 55 included reviews. This form was developed ad hoc for this review, but was based on tools previously used by others. Nine categories, derived from other reviews of reviews [19,22], pertained to characteristics of the focus and methods of each review (general and specific behavior focus, target population, intervention setting, type of review, time span, number of studies included, criteria for study design and outcome measures). One category was used for recording general results with respect to effectiveness, such as overall effect sizes or general statements. The other 17 categories addressed results with respect to effective elements of programs, participants or studies. This level of specificity was chosen to maximize learning about characteristics associated with effectiveness. Seven of these categories, which are all discussed in this review, pertained to elements of programs: focus/goal, development, content, methods, facilitator, components, and intensity. The remaining 10 categories pertained to elements of participants (e.g. gender, pre-test risk behavior) or studies (e.g., type of study design, length of follow-up). The three main categories of effective elements (programs, participants and studies) and specific elements within these categories (e.g., for program characteristics: goal, development, et cetera) are commonly used in data extraction forms of systematic reviews [e.g., see [40,75]]. Due to the length of this paper we will not discuss the results for elements of participants or studies in full but will only address them when they are relevant to results for program elements.

Results and statements about effectiveness and effective elements were recorded in the appropriate categories as specifically as possible, often by literally quoting the review author. In addition, the results of each review were summarized using the symbols +, -, 0 and ? for respectively a positive, negative, null or unclear contribution of the element to effectiveness. This 'shorthand notation' facilitated tabulation, whereas the underlying extensive information warranted preservation of details. This process resulted in a 195-page summary document and an 80-page document with tables.

The first author extracted all data and conferred with the third author in case of doubt about interpretation or recording of a specific result; this was the case with 20 reviews. The third author also read six reviews (11%) and checked all data extracted from these reviews; only a few disagreements were found and these were discussed until a unanimous decision was reached.

### Quality rating

The included reviews were rated for methodological quality using the Quality Assessment Tool for Reviews. This tool was developed by the Effective Public Health Practice Project and has been used in several reviews of reviews [19,22,24]. It comprises the following seven criteria, which are all awarded one point, with a maximum score of 0 to 7: a) statement of the search strategy; b) comprehensiveness of the search; c) description of relevance criteria; d) some quality assessment of primary studies; e) comprehensive quality assessment of primary studies; f) integration of findings; and g) adequacy of the reported data to support the review's conclusions. Quality was rated by two raters in a staged manner. First, the independent ratings of 13 reviews were compared (inter-rater reliability overall: kappa = 0.639, p < .001), and disagreements were discussed and resolved. Then, the remaining reviews were rated independently, and compared, and any disagreements were discussed until all scores were unanimous. Reviews were rated strong if they met six or seven of the criteria, moderate if they met four or five, and weak if they scored three or less. Strong reviews tend to be systematic, and weak reviews tend to be traditional narrative reviews. In addition to quality criteria d and e, which are quite general and only ask whether reviews assessed the quality of primary studies, we recorded which specific methodological inclusion criteria were applied in reviews [see Additional file 1 – Table S1].

### Analysis

For each program element, the results of included reviews were compared, first within each domain, then across domains. Following procedures used in other reviews of reviews [21,22,24], only the results of strong and moderate reviews were considered for statements about effective elements. We considered a program element to be effective in a particular domain if it was labeled as such in at least one strong or moderate review from that domain and, in case of multiple reviews, if the overall conclusion was positive. If strong and/or moderate reviews in one domain had conflicting results (e.g., positive versus null results), we attempted to reach an overall conclusion by examining the methodology of the reviews (e.g., did follow-up periods or criteria for effectiveness differ between reviews?) and giving the highest weight to the review with the highest quality score, the strictest methodological criteria, and the clearest and most narrowly defined operationalizations; if no overall conclusion could be drawn the evidence was considered to be inconclusive.

Additionally, it was examined whether the results would be the same when using an alternative analytical approach, which was derived from others [21]. In this second type of analysis, the strength of evidence is rated as sufficient, tentative or insufficient based on explicit rules. The evidence is sufficient if it is based on conclusions in at least one strong review from that domain and if there are no conflicting conclusions between strong reviews. The evidence is tentative if it is based on at least one moderate review or if the conclusions of strong reviews conflict (e.g., positive versus null results). If moderate reviews have conflicting conclusions, the evidence is considered to be insufficient. The main differences between the two types of analysis are that the second type strictly distinguishes between strong and moderate reviews and relies on a strict rule for handling conflicting results, whereas the first type relies more on interpretation of conflicting results. Hence, the first type is called interpretation-based and the second is called rule-based.

The results of weak reviews were deemed to be too questionable for conclusions about effective elements. However, in light of the focus of this review on similarities across domains, they were included in a supplementary way. Specifically, if a particular element had evidence from strong or moderate reviews in at least one domain, the results of weak reviews in other domains were explored and treated as a suggestion that the element might be effective in these other domains.

## Results

### Characteristics, relevance and quality rating of included reviews

[Additional file 1 – Table S1] gives an overview of characteristics of the 55 reviews. The reviews are categorized by behavior focus, and within these categories, by quality rating and publication year.

In addition to – or instead of – a preset focus on one or more behaviors, some reviews focused on specific populations (e.g., young adolescent girls [39]), intervention types (e.g., peer education [26,37,69]) or even specific programs (e.g., Life Skills Training [47]). Such specific foci are reported in Table S1.

All reviews included school-based programs (not reported in Table S1), and 23 of them entirely focused on programs in this setting, among which 15 in the substance abuse domain. Substance abuse prevention and sex education are usually implemented in secondary schools (junior high and/or senior high) and may also include the upper elementary grades 5–6. This corresponds with the age range most frequently stated in reviews: 11–18 years. Many nutrition reviews also included younger elementary-aged children.

The number of included studies differs widely across the reviews (3–144 studies) and appears to be largely due to differences in review focus (e.g., specific program type) and strictness of methodological inclusion criteria. For reviews that provided sufficient information about studies, we recorded in Table S1 how many of the included primary studies met our relevance criteria (targeted behaviors, secondary-school-age, school-based educational intervention). In the nutrition domain, some reviews included only one relevant study, as most nutrition programs target elementary students. For these reviews, only the results of this one study were recorded. In the other domains, the number of relevant studies was much larger, and often all studies were relevant.

Except for a review about sexual knowledge [67], all reviews applied behavioral criteria to determine program effectiveness. Many reviews also addressed effects on psychosocial determinants, and in the sexuality domain one third of reviews examined results for biological outcomes such as pregnancy.

As for the quality rating, 36 reviews (65%) were rated strong, 6 moderate (11%), and 13 weak (24%). Weak reviews generally did not report methodological inclusion criteria, whereas strong reviews did. Criteria used most frequently pertained to study design and outcome measure; other criteria were much less frequently applied, e.g. for equivalence of groups, minimal follow-up period, or reporting of all outcomes. The inclusion criteria differed markedly, even between strong reviews. Many strong reviews subjected the included studies to additional quality rating. Fifteen reviews applied meta-analytic techniques (mostly in the substance abuse and sexuality domains, not reported in Table S1) and nearly all of them had a quality score of 7.

### Effect sizes and general statements about effectiveness

Qualitative statements about the occurrence or magnitude of behavioral effects were cautiously positive in most reviews. Only very few reviews reported overall absence of effects and none reported overall negative effects. There do not appear to be clear relationships between type of statement and behavioral domain or review quality. The quantitative results of meta-analyses and reviews, expressed in effect sizes (ES), odds ratios (OR) or percentage reductions, are in line with the above mentioned qualitative statements in the reviews: in light of Cohen's [81] classification of ES as small (.20), medium (.50) or large (.80), many ESs reported in reviews were statistically significantly different from zero, explaining positive statements, but most can be considered small, explaining reservations.

In the *substance abuse *domain, average ESs reported for tobacco use ranged from -.02 [[41]: for the total set of non-interactive programs] to .29 [[32]: for life skills programs evaluated within 12 months after end of the program], with most meta-analyses reporting ESs between .10 and .18 [32,40,41,43,45]. Botvin and colleagues [29,47,53] reported typical reductions of 30–50% for social influence programs and 40–80% for life skills programs. A review of long term (> 2 years) tobacco outcomes reported a mean reduction of 11.4% in the percentage of baseline nonusers who initiated smoking [44]. For alcohol use, meta-analyses [40,41] and reviews [29,44,47,53] have reported ESs and percentage reductions of the same magnitude as for tobacco use.

In the *sexuality *domain the results vary per outcome measure examined and per review. Statistically significant positive effects have been reported for condom use (ES = .07 [56]; OR = .66 [58]). For birth control, one meta-analysis that included non-controlled studies found statistically significant positive effects (ES = .27 [61]) but a meta-analysis with stricter study design criteria did not [57]. Of five reviews that examined sexual activity, frequency or number of partners, two reported statistically significant positive effects (both ES = .05 [56,59]), whereas the other three did not [57,58,61]. No effects were found on diagnosis with STD [56,58]. As for pregnancy, the meta-analysis that included non-controlled studies reported a positive effect (ES = .15 [61]), whereas one with stricter criteria found no effect for females and a negative effect for males (OR = 1.54 [57]).

In the *nutrition *domain, statistically significant positive effects have been reported for intake of fat (OR = 2.19 [75]) and fruit and vegetables (increase of .30 to .99 servings per day [72]). One intensive high school intervention even increased daily servings of fruit and vegetables by over 2.5 [74,76].

ESs reported for psychosocial determinants are usually larger than those for behavior. In the substance use domain, a meta-analysis [41] reported an average ES of .38 for knowledge, .26 for attitude and .24 for skills for programs with much peer interaction. A tobacco-specific meta-analysis [32] reported comparable ESs for knowledge (.53 to .19, depending on the follow-up interval), attitude (.22 to .10), and skills (.22 to .09). In the sexuality domain, the following ESs have been reported: .41 for knowledge [67], .30 for condom use skills and .50 for condom negotiation skills [56].

### Effective elements of programs

The results for the various categories of program elements are presented in [Additional file 1 – Supplemental Tables S2-S8] and are discussed in separate paragraphs below. As stated in the Methods section, the analysis focused on results of strong and moderate reviews; weak reviews were only used for supplementary purposes in the absence of stronger reviews. The elements are italicized in the text below to enhance combined reading of text and tables, and elements that are considered effective in all three domains are marked bold in the text and tables. In light of the large number of elements that have been examined in the reviews and our focus on similarities across domains, the tables only include aspects that have been examined in at least two domains.

### Program focus or goal

As shown in [Additional file 1 – Table S2], several strong reviews in the nutrition and sexuality domains concluded that programs with a *specific behavioral focus *(e.g., fruit consumption, condom use) are more effective than programs that discuss general nutritional or sexuality issues; supplementary, a comparable statement in one weak substance abuse review was that programs should be tailored to specific substances [52].

The issue of *abstinence goals *has been addressed by strong reviews in the sexuality and substance abuse domains. Not one sexuality review stated positive conclusions about the effectiveness of abstinence-only programs, which portray abstinence from sex as the only or very best prevention option and usually do not discuss contraception, and one even reported negative effects [63]. In contrast, one strong sexuality review [61] reported positive effects of programs that do discuss contraception (abstinence-plus or safer sex programs). Comparatively, in the substance abuse domain, one strong review cautioned that the goal of harm reduction or prevention of abuse may be more effective than a goal of abstinence or delayed use, at least for youth who already use [35].

### Program development

In the substance abuse, sexuality and nutrition domains there is broad consensus among strong reviews that ***theory-based ***programs produce better effects than non-theory-based programs [see Additional file 1 – Table S3], although some reviews did not find obvious differences [42], only found a contribution of theory in univariate and not multivariate analysis [56] or stated that the exact contribution of using theory is unclear [26]. With respect to specific theories, strong reviews in the substance abuse [36,40] and nutrition [77] domains made special reference to Bandura's *social cognitive theory*; supplementary, a weak review in the sexuality domain stated that the evidence for using this theory is tentative but not yet convincing [70].

*Addressing behavioral determinants *was reported to be an effective element by a strong nutrition review [77] and a moderate sexuality review [66]; supplementary, weak reviews in the substance abuse domain had the same conclusion [52-54]. Three other characteristics of program development were stated to be important for enhancing effects, but each only in one or two domains: *needs assessment *among the target group, *participant involvement *in program planning and implementation, and *pretesting*. The evidence for the second element involved only a supplementary weak review in the substance abuse domain [54], and the evidence for the third was mixed, as a meta-analysis in the sexuality domain reported that stated use of pretesting was not related to the effect size for condom use [56].

The issue of *tailoring interventions to the culture *of the target group was addressed by several strong or moderate reviews in the substance abuse domain and a moderate review in the sexuality domain. The sexuality review had positive conclusions [66], as did most substance abuse reviews [33,41,47]. However, the substance abuse review with the strictest criteria reported this issue to be unclear because no high-quality study had compared culture-specific interventions with standardized interventions [31]. In the nutrition domain, this issue was only addressed by a supplementary weak review, which stated the issue to be unclear and in need of further research [79]. *Tailoring to cognitive ability or age *has been examined by three strong reviews, which cover all three domains. The sexuality [65] and nutrition [77] reviews reported favorable results, but again, the review in the substance abuse domain applied the strictest criteria and reported inconclusive results because of a lack of high-quality comparison studies [31].

### Program content

[Additional file 1 – Table S4] presents the results for elements of program content. Since many elements were mentioned in the reviews, we included headings to indicate that there may be some similarity between elements.

#### Knowledge, risk, attitude

Health education programs in all domains usually include information about health consequences and prevention methods. In all domains a *knowledge-only approach *was reported to have no effect on behavior, but in the sexuality domain this involved only a supplementary weak review [29]. Some authors commented that this approach has hardly been tested rigorously [31] or only with traditional, non-engaging methods [51]. In the sexuality domain, a strong and a moderate review stated that accurate, *factual information *is an element of effective interventions [60,66]; supplementary, this was also reported in a weak substance abuse review [54]. The results of two strong sexuality reviews for *enhancing perceived risk *were mixed [58,65]; in the substance abuse domain, the related issue of fear arousal was reported to be ineffective by a moderate review [47]. Several other elements were each addressed in only one domain and are therefore not included in Table S4 nor further discussed here.

#### Social influences

***Social influences ***have been addressed in all domains, especially in the substance abuse domain where the social influences approach has been widely prevalent for decades. In all domains, strong reviews stated that this approach is effective, although reservations were reported in one tobacco review [31] as the largest and most rigorous study found no evidence of a sustained effect on smoking prevalence. While the social influence approach entails several components [see [51]], two components have received most attention in the review literature: reinforcing or changing social norms (e.g., correcting overestimations of peer smoking) and training in recognizing and resisting peer, media and other influences (e.g., learning to negotiate safer sex). In all domains, strong reviews reported the first component, addressing ***social norms***, as an effective element. In the nutrition domain attention to norms does not seem to take the form of normative feedback but rather of building normative support for desired changes and for creating a more supportive school or community environment [77]. The second component, *resistance skills training*, was not addressed in nutrition reviews and had inconsistent results in other domains. There is some evidence that this element may only be effective in conjunction with normative education or with a rationale or motivation for refusal and may even be counterproductive when used alone [28]. This latter review [28] reported that resistance skills training is only effective if it is behavior-specific.

#### Skills

In all domains, training of *skills *was generally reported to be effective. Although the types of skills were not always specified or seemed to vary, the following similarities were observed. In the nutrition and sexuality domains, some strong reviews mentioned *domain-bound practical skills*, such as food preparation or condom use skills.

In each domain, ***cognitive-behavioral programs ***have been found effective in one or two strong reviews. Although not all authors used the same terms or were clear about what this approach entails exactly, we included this element to refer to statements about the importance of addressing both motivations and cognitive and behavioral skills. In the nutrition domain, one strong review stated that effective behaviorally focused curricula address cognitive, affective and behavioral aspects [77]. In their meta-analysis of tobacco outcomes of psychosocial programs, Hwang and colleagues [32] used a narrower definition of cognitive-behavioral programs. They distinguished social influence, cognitive behavioral, and life skills modalities. Cognitive-behavioral programs were those that included the social influence approach "plus at least two cognitive skills such as problem solving, decision making, assertiveness, self-control, and/or other coping skills. Life skills programs included the defined aspects of the social influence and cognitive-behavioral modality programs plus at least one affective skill such as self-confidence, values clarification, and/or generic social skills".

*Life skills training *can be regarded as a specific type of cognitive-behavioral program, one that addresses self-management and social skills (decision-making, anxiety management, communication, assertiveness). Strong reviews in the substance abuse domain reported that this training enhances the effects of a social influence approach on tobacco and alcohol use. Life skills training has only been tested in the substance use domain, and only in combination with a social influence approach. However, in the sexuality domain some strong and moderate reviews seem to refer to similar skills when stating the importance of coping, communication, and negotiation skills [58,60,62,65,66], not reported in Table S4].

### Program methods

Statements about effective methods were relatively scarce in the reviews [see Additional file 1 – Table S5]. In the substance abuse domain four strong reviews consistently reported *interactive methods *to be effective; supplementary, weak reviews in the sexuality and nutrition domains mentioned specific examples of interactive methods (discussion and role-play). Tobler and colleagues [40,41], who provided the strongest evidence for interactive methods in large meta-analyses in the substance abuse domain, stated that interaction should be between students, not so much between student and teacher.

In both the nutrition and sexuality domains, *having students personalize information *was identified as an effective element in one strong or moderate review. Four other elements of program methods had evidence from one or two strong reviews in one domain, but had been examined by only weak reviews in another domain. The results for these elements were consistent across these domains (the domain named first in parentheses had evidence from a strong review): a *traditional, didactic style *('lecture') is reported to be ineffective (nutrition, substance abuse), whereas it is effective to use *multiple channels *(sexuality, multiple behaviors),* active, experiential methods *such as experiments and taste testing (nutrition, substance abuse), and *cognitive-behavioral skills training *(sexuality, substance abuse). According to one review [47], the latter training consists of: instruction and demonstration, behavioral rehearsal with role play, feedback on each student's performance, social reinforcement, and extended practice through behavioral 'homework' assignments. Several other methods have only been reported in a single domain and are thus not included in Table S5 nor discussed here (e.g., modeling, goal-setting).

### Program facilitator

The impact of the type of program facilitator on program effectiveness has had most attention in the domains of substance abuse and sexuality [see Additional file 1 – Table S6]. Especially in the substance abuse domain, many types of facilitators have been examined (not shown in Table S6).

Only *peer leaders *and *teachers *have been examined in more than one domain. The evidence conflicted between the nutrition and sexuality domains, as a strong nutrition review reported favorable results for the use of peer leaders [72], whereas three strong sexuality reviews did not find evidence for a differential impact of the type of facilitator [55,56,59]. In the substance abuse domain, the results of strong and moderate reviews were mixed. Both peer leaders [45,47] and teachers [47] have been involved in effective programs and several meta-analyses and reviews that analyzed the contribution of the type of facilitator to ES did not find overall significant differences between these facilitator types [34,35,41]; however, some reported results favoring peers over teachers, either overall [43] or for a particular intervention type [34,40] or measurement period [36]. A meta-analysis of studies comparing implementation of the same program by *peers versus teachers *reported that peers have shown better effects, but only in the short term and not at 1- or 2-year follow-up [37]. However, in light of variations in effects and lack of high-quality studies, this review did not conclude that implementation by peers is better. Also, a recent tobacco review [31] stated that not one comparison study was of high quality. Our overall conclusion for the substance abuse domain is that there are some indications that peers may have better effects than teachers, but the evidence is yet inconclusive and not one type of facilitator has generally proven to be more effective than another. There was one element of the facilitator that was consistently reported by strong reviews in all domains to have a positive contribution to effectiveness: ***facilitator training***.

### Program components

[Additional file 1 – Table S7] presents the results of reviews with respect to program components. The term 'component' is used here to refer to different approaches to behavior change (education, environmental change) or the inclusion of different settings (school, family, community). We paid extra attention to reviews with a specific focus on schools, and we were especially interested in the added value of school-wide, family and community components in addition to the usual classroom education approach.

Strong reviews in all domains were consistently positive about the effectiveness of ***programs with multiple components***, except for one sexuality review with null results but unclear operationalization [57] and one tobacco review that reported positive effects only for the long term [32]. The element of multiple components includes statements about the (better) effects of multi-component programs in general, about specific multi-component programs and about combinations of specific components.

Drawing overall conclusions about specific components is more difficult because reviews varied as to the specificity of their statement, the operationalization of components, and the criteria for assessing effectiveness (e.g., are direct comparisons necessary?). For instance, several reviews distinguished family from community components, whereas others included all family, media and community mobilization activities under the heading of community components. In light of these differences between reviews, the conclusions below about specific components should be regarded as tentative.

Programs with *school-wide change and family or community components *have been reported by strong reviews to be effective, but have only been examined in the substance abuse and nutrition domains. Strong reviews in the substance abuse and sexuality domains made positive statements about *community interventions*, and these were supplemented by a weak review in the nutrition domain; however, the strong alcohol review by Foxcroft and colleagues [33] referred more to hypotheses about cost-effectiveness than to actual evidence. The added value of *community adjuncts to classroom interventions *is convincing in the nutrition domain but was not examined in the sexuality domain. In the substance domain, several strong reviews and meta-analyses had positive conclusions, but their operationalizations or statements were general and included also family activities [32,36] or life skills modalities [31].

The evidence for *school-wide activities *is consistently positive in the nutrition domain (foodservice); supplementary weak reviews in the sexuality domain were also consistently positive (school health clinic with family planning services), but weak reviews in the substance abuse domain were not (school drug policies).

There is some evidence from strong reviews in all domains that including *parents or families *is effective; however, in the substance abuse domain this may apply only to high-risk youth, and in the nutrition domain only to elementary-aged children [77].

In the nutrition domain one strong review examined policies that impact on accessibility of products. *Price regulation *has been found effective in this domain [78]; this was also reported for tobacco and alcohol by two weak substance abuse reviews [52,54].

All in all, there is some evidence in all domains that multi-component programs with school-wide, community and/or family components can be effective or can be more effective than curricular interventions, but the added value of such components is unclear.

### Program intensity

Table S8 [see Additional file 1] lists the review results with respect to program intensity and duration. It should be noted that it is not always clear what authors mean when using these terms. The more narrowly defined term of *number of sessions/hours *was addressed by strong reviews in all domains. Only reviews in the nutrition domain consistently reported a positive association with outcomes ('more is better') [74,76,77]. In the sexuality domain, the results appear to differ per type of review: three narrative reviews reported such an association [55,60,62], whereas two meta-analyses did not [58,59]. In the substance abuse domain, one review and one meta-analysis did not find clear evidence that more is better [35,41], whereas another meta-analysis did, but only for interactive programs and not for non-interactive programs [40].

Several strong or moderate reviews identified a specific minimum number of sessions/hours required for producing effects, and the numbers were comparable across domains: 8 hours for sexuality programs [60] and 10 sessions for substance abuse [48] and nutrition programs [74], although one nutrition review considered 10–15 sessions insufficient [77]. These numbers are in accordance with effects reported in one strong and one moderate substance abuse review about specific programs [38,47], but another review stated that recent substance abuse studies tend to recommend fewer sessions, specifically 4, 5 or 8 [35]. However, in light of the results already discussed, the evidence that a larger number of sessions enhances effects is only consistent in the nutrition domain. The same conclusion can be reached for the less well-described terms of *intensity *and *duration*.

The issue of *booster sessions *has mainly been examined in the substance abuse domain, except for one strong sexuality review with positive results [65]. In the substance abuse domain, the results of strong reviews were mixed. Of two strong tobacco-specific reviews, one concluded that boosters enhance long-term effects [44], but our recalculations of the presented data led us to question this conclusion; the second review had unclear results [43]. One broader substance abuse review reported benefits of boosters for behavior maintenance [35], while another did not find conclusive evidence and stated that boosters may increase effects for some programs but not for others [36]. All in all, this issue remains inconclusive.

## Discussion

### Similarities across Domains

This review of reviews examined effective elements of adolescent health promotion programs in three behavioral domains – substance abuse, sexual behavior and nutrition. We specifically focused on similarities across these domains, and indeed, we identified many similarities. The results are discussed here in light of the two types of analysis that have been explained in the Methods section: an interpretation-based method and a rule-based method. Based on our interpretation-based examination of the evidence that is currently available from strong and moderate reviews, five elements were identified to be effective in all domains. These five elements have evidence from at least one strong review in each domain:

a) use of theory, with specific reference to social cognitive theory

b) addressing social influences, especially social norms

c) addressing cognitive-behavioral skills

d) training of facilitators

e) including multiple components.

When using the rule-based method of analysis, the results are similar: all five elements have at least tentative evidence in all domains. Elements b, c and d even have sufficient evidence in all domains; elements a and e have tentative evidence in one or two domains due to conflicting results between strong reviews in these domains (positive versus null or unclear results). Using the rule-based method, no other elements were identified as having sufficient evidence in all three domains, but two additional elements had at least tentative evidence for a positive contribution to effectiveness in each domain:

f) parent involvement

g) a larger number of sessions.

These two elements were not identified as similar across domains with the interpretation-based method of analysis, since we found the evidence in at least one domain to be inconclusive due to conflicting results between strong reviews; in the rule-based method such conflict leads to the conclusion that the evidence is tentative. The different results of the two methods of analysis for these two elements can thus be explained by the different approaches to handling conflicting results.

In addition to the above elements, which had evidence from strong or moderate reviews in each of the three domains, several other elements also tended to have similar results across the three domains, but their evidence involved only weak reviews in one or two domains. Although weak reviews were not included in the analysis, they were used for exploring whether there is any indication that a particular element might be effective in a particular domain. The following elements had similar results across all domains; domains with strong or moderate reviews are given between parentheses:

h) a focus on specific behavior (sexuality, nutrition)

i) addressing behavioral determinants (sexuality, nutrition)

j) a knowledge-only approach (ineffective element; substance abuse, nutrition)

k) use of interactive methods (substance abuse).

In addition to the above elements, the results for many other elements were comparable across at least two of the three domains. We did not find one element for which the results indicated opposing directions of influence between domains (e.g., a positive contribution to effectiveness in one domain and a negative contribution in another domain). In cases where the results were not similar across domains, this was usually because results in one or more domains were unclear or indicated null findings, whereas those in other domains indicated a positive contribution to effectiveness.

The results of the present review are fairly similar to those of other systematic reviews of reviews that examined the domains of substance abuse and sexuality separately and that included only high-quality reviews [19,27], suggesting that the results for these domains are robust. This review adds rigor and specificity to the general observation in several reviews that effective elements in the domains of substance abuse and sexuality appear to be similar [e.g. [17-19,29,66] and extends this observation to also include the nutrition domain. In contrast to the present review, these reviews did not examine the issue of similarity systematically or in detail.

Perhaps more importantly, our results are largely comparable to, and in some cases more specific than, those of a review of reviews that specifically focused on similarities across multiple domains [16]. That review examined partly different domains (substance abuse, risky sexual behavior, school failure, and juvenile delinquency and violence), included a smaller and different set of reviews (35 narrative reviews that explicitly discussed common features of effective programs) and used a somewhat different review methodology (determining the percentage of reviews that mentioned an element as consistently effective). In that review [16], nine elements of effective programs were identified, which were claimed to reflect general principles that transcend specific content areas. Seven of these elements coincide with the ones identified by us, although some tend to be formulated in more general terms than ours. These seven elements and, between brackets, the corresponding letters from our list, are: theory-driven [a]; socio-culturally relevant (address cultural norms and beliefs) [b, i]; varying teaching methods (skills-based component, active and interactive format) [c, k]; providing opportunities for positive relationships (parent-child communication, peer influences) [b, f]; well-trained staff [d]; comprehensive (multi-modal, multiple settings) [e]; and sufficient dosage [g]. Two of the elements they identified are not represented in our own set of eleven elements: appropriate timing and inclusion of outcome evaluation. The issue of outcome evaluation was not considered relevant for the present review, as it is an aspect of studies rather than programs. The issue of appropriate timing has to do with tuning interventions to student characteristics such as age, cognitive and social development and experience with the risk behavior. This issue is generally recommended in health promotion theory [82,83], and indeed, tailoring to age was reported to be effective by strong sexuality [65] and nutrition reviews [77] in this paper. However, we did not include it in our empirically-based list of effective elements because in the substance abuse domain it was reported to be unclear due to absence of high-quality comparison studies [31]. One element from our own list, a focus on specific behavior, is not represented in the list from the other review [16]. Unfortunately, due to the limited reporting of results in that review [16], we cannot examine the causes for this difference. Possibly, the issue of behavioral focus may only be relevant for certain domains or may have been overlooked in certain domains.

### Implications for Practice

Researchers and practitioners in the three domains can use the effective elements identified in this review, and especially the ones that are similar across domains, as guidelines for developing and improving their adolescent health promotion programs. They can also look beyond the boundaries of their own domain to generate ideas for programs or research from results in other domains.

The fact that another multiple-domain review [16] found comparable effective elements while examining partly different domains (also school failure, juvenile delinquency and violence) suggests that the effective elements may transcend broadly to other content areas. In fact, the effective elements pertaining to program development (use of theory, addressing determinants) appear to be applicable universally, as they are general recommendations from health promotion planning models and quality assurance procedures such as PRECEDE-PROCEED [82], intervention mapping [83] and Preffi [84].

The finding that several effective elements are comparable across the three targeted domains indicates that integrative programs can address these domains with the same program characteristics. This is important in light of the recent interest in multiple health behavior research and its potential implications for integrative interventions [6,11,85]. The results will be used for guiding the development of our own integrative program. The effective elements pertaining to program content – address not only information, but also social influences and cognitive-behavioral skills – fit well with those of a previous review that assessed similarities between behavioral determinants across the same three domains [10]. In that review the following determinants were found to be important for all domains: attitudinal beliefs about immediate gratification and social advantages, social norms, modeling behavior and resistance skills. Together, both that review and the present one provide sufficiently valid input for the development of an integrative program that addresses all three domains.

### Limitations

Given our broad focus on several health-related behaviors and the already extensive body of knowledge in each domain, we applied a review-of-reviews approach, an approach that has gained acceptance in recent years [e.g., [16,19,21-24,27,35,36]. Although the search strategy was comprehensive, it is possible that we may have missed relevant reviews. However, it is not very likely that these reviews would have discussed different sets of primary studies and would have led to different conclusions.

A limitation of the review-of-reviews approach is that it relies on 'second-hand' information and is potentially vulnerable to the interpretive and conceptual biases of previous reviewers [16]. We attempted to limit these biases as much as possible by using a systematic review methodology, by assessing the quality and relevance of each review and relying on reviews of high to moderate quality, by carefully categorizing the results without generalizing too much, and, in case reviews had differential results, by attempting to examine the causes of the differences. We also attempted to check the results of reviews if sufficient information was provided.

Perhaps we would have identified more similarities across domains if we had combined aspects and findings into broader categories. We used a conservative categorization process and were reluctant to generalize findings, because the operationalization, interpretation or analysis of aspects seemed to differ between reviews or were sometimes unclear.

Two-thirds of the included reviews had a high quality score of 6 or 7. In line with other reviews of reviews [21,22,24] we included only strong and moderate reviews in the analysis. Furthermore, we used two methods for analyzing the results and especially for dealing with conflicting results between reviews: one method focused on interpretation of differences and the other set a strict rule. The conclusions based on these two methods were fairly similar. Weak reviews were excluded from the analysis but were used in a speculative way: for elements that had evidence from strong or moderate reviews in at least one domain, the results of weak reviews in the other domains were used to give any indication or suggestion of effectiveness in these other domains.

The methodological aspects assessed in reviews most often pertained to study design, appropriateness of allocation procedures, comparability of groups, validity of assessment and attrition, but only a few reviewers examined additional aspects such as quality of implementation. The strictness of inclusion criteria and assessment of methodological quality varied widely, even among high-quality reviews. Although meta-analyses in several domains reported that effect sizes did not vary with the design or quality of studies [34,40,56-58,72], reviews with the strictest methodological criteria (e.g., accepting only high-quality comparison studies) generally appeared to have more cautious conclusions than reviews with less strict criteria. Reporting the specific criteria applied by reviewers appears to be a valuable addition to the Quality Assessment Tool for Reviews. For reviews of primary studies, the Cochrane Collaboration Handbook [86] and others [87] recommend using the Quality Assessment Tool for Quantitative Studies, which is also developed by the Effective Public Health Practice Project, Canada.

## Conclusion

A multitude of effective elements of school health promotion programs has been identified in literature reviews in the domains of substance abuse, sexuality and nutrition. Many effective elements are similar across at least two domains. Based on strong reviews in all three domains, five elements were found to be similar across the three domains: use of theory; addressing social influences, especially social norms; addressing cognitive-behavioral skills; training of facilitators; and including multiple components. Two additional elements had at least tentative evidence of effectiveness in all domains when using a rule-based method of analysis but had inconclusive evidence in at least one domain when using an interpretion-based method of analysis: parent involvement and a larger number of sessions. For four additional elements, the results were comparable across the three domains but they are more speculative, as in one or two domains these elements had only been examined by weak reviews. Three of these elements have a positive contribution to effectiveness (specific behavioral focus; addressing determinants; interactive methods), whereas the fourth (knowledge-only approach) was considered ineffective. The results suggest that an integrative program that addresses the three domains seems feasible and could be efficient. The five elements with evidence from strong reviews in each domain are likely candidates to include in such a program.

## Competing interests

The authors declare that they have no competing interests.

## Authors' contributions

LP conducted the literature searches, the selection of reviews, the data extraction, the quality rating and the data analysis, and drafted the manuscript. GK participated in the design and coordination of the study. GTD participated in the design and coordination of the study, the formulation of search criteria, and the selection of reviews, and checked the data extraction and quality rating. GB participated in the design of the study. TP conceived of the study and participated in its design and coordination. All authors read and approved the final draft.

## Pre-publication history

The pre-publication history for this paper can be accessed here:



## Supplementary Material

Additional file 1 **Supplemental Tables S1-S8**. Table S1 contains information about the characteristics of the included reviews, Tables S2-S8 contain the results of the reviews for the various categories of program elements Click here for file
