# Supplementary material for: Effective elements of school health promotion across behavioral domains: a systematic review of reviews
Source: BMC Public Health. 2009 Jun 12;9:182. doi: 10.1186/1471-2458-9-182 (PMC2702385; doi:10.1186/1471-2458-9-182)
Supplement: Additional file 1 — Supplemental Tables S1-S8. Table S1 contains information about the characteristics of the included reviews, Tables S2-S8 contain the results of the reviews for the various categories of program elements [file 1471-2458-9-182-S1.doc]

**Table S1. Characteristics of included reviews, categorized by behavior focus**

| **Reference^a^ (quality)** | **Behavior focus^b^** | **Time span^c^** | **# Studies**  **(# relevant)^d^** | **Age range^e^** | **Methodological inclusion criteria^f^** |
| --- | --- | --- | --- | --- | --- |
| **Multiple domains** | | | | | |
| 26 Harden 1999 (7) | Peer programs. Tob, Sex, other | Up to 1998 | 49, 12 sound (5) | 11-24 yrs | Prospective controlled design, equivalent groups, reporting of all data for all groups. |
| 27 Lister-Sharp 1999 (7) | Health promoting school approach. Tob, Sex, Nut, other | No limits  (incl =1979-1998) | 12 (6) | 5-16 yrs | One-group pre-post or controlled design, health-related outcomes |
| 28 Herrmann 1997 (6) | Refusal programs. Tob, Alc, drugs, Sex | 1974-1994 | 33 | Child, Adol | None stated |
| 29 Botvin 1995 (2) | Tob, Alc, drugs, Sex (aids, std, pregnancy) | NR | 141 r | Child, Adol | None stated |
| 30 Jason 2002 (1) | Tob, Alc, drugs, Sex, Nut, other | NR | 50 r | Child, Adol | None stated |
| **Substance abuse** | | | | | |
| 31 Thomas 2006 (7) | Tob | 1966-2005 | 94 (54 in analysis, 23 high-quality) | Child (5-12 yrs), Adol (13-18 yrs) | RCT, tobacco use measure for baseline nonusers, minimal follow-up of 6 months after program end. Additional quality rating. |
| 32 Hwang 2004 (7) | Psychosocial programs. Tob | 1978-1997 | 75 | Grade 6-12 | Control group had no psychosocial program, pre-post measures |
| 33 Foxcroft 2003 (7) | Alc | Up to 2001 | 56 | < 25 yrs | RCT, matched pre-post or interrupted time series design; measures of alcohol use or related problems. Additional quality rating. |
| 34 Gottfredson 2003 (7) | Alc, drugs (not Tob-only) | NR | 94 | School-age | Control group with no or minimal intervention, behavioral outcome |
| 35 McBride 2003 (7) | Tob, Alc, drugs | 1997-2001. Reviews 1990-2001 | 5 primary studies, 11 reviews | School-age | Primary studies: (quasi-)experimental design, pre-post measures of behavior, positive effects, discussed methodological issues. Reviews: systematic, applied methodological inclusion criteria |
| 36 Cuijpers 2002 (7) | Tob, Alc, drugs | NR, incl = mostly 1990s | 27 (27) | School-age | Meta-analyses comparing program types. Mediator studies. Studies comparing program components. |
| 37 Cuijpers 2002 (7) | Peer-led vs adult-led. Tob, Alc, drugs | NR, incl = 1981-1995 | 12 (12) | School-age 11-18 yrs | Prospective studies comparing peer- and adult-led program. |
| 38 Sussman 2002 (7) | Towards No Drug Abuse. Tob, Alc, drugs, violence | 1994-1998 | 3 (3) | Grade 10-11 | None stated, all studies were RCT |
| 39 Blake 2001 (7) | Tob, Alc, drugs | 1980-2000 | 32 (11) | Adol girls (primary & secondary age) | Female-specific results, no design criteria |
| 40 Tobler 2000 (7) | Tob, Alc, drugs | 1978-1998 | 144, 93 high-quality | School-age | Controlled design, pre-post drug-use measures. Additional criteria for high quality subsample. |
| 41 Tobler 1997 (7) | Tob, Alc, drugs | 1978-1990 | 90, 56 high-quality | Grade 5-12 | Controlled study with pre-post drug-use measures. Additional criteria for high quality subsample. |
| 42 Foxcroft 1997 (7) | Alc | 1966-1995 | 33 | 8-25 yrs | (Quasi-)experimental, pre-post measures of alcohol use or related incidents. Additional quality rating. |
| 43 Rooney 1996 (7) | Social or peer-type programs. Tob | 1974-1991 | 90 | Grade 6-12 | Control group, measure of tobacco use |
| 44 Skara 2003 (6) | Tob, Alc, drugs | 1966-2002 | 25 (25) | < 21 yrs | (Quasi-)experimental design; at least 2-yr follow-up; measure of tobacco use incidence or prevalence |
| 45 Posovac 1999 (6) | Peer-based programs. Tob, other | 1978-1997 | 22 Tob programs | Average age 12.5 yrs | Control group, enough information to calculate effect sizes |
| 46 Werch 2001 (5) | Stage-based programs. Alc | About 1990-2000 | 5 (3) | NR. Included = Grades 5-9 | None stated, all studies were RCT |
| 47 Botvin 2000 (5) | Life Skills Training. Tob, Alc, drugs | About 1980-2000 | About 9 (about 9) | Middle/junior high school | None stated |
| 48 Dusenbury 1997 (5) | Alc, drugs (incl Tob but not Tob-only) | NR | 23 (21) | Primary and secondary age | Pre-post control group design, substance use outcome measure, peer reviewed |
| 49 Hittner 1998 (4) | Alc | 1980-1996 | 36 (31) | Child, Young Adol | Alcohol misuse outcome |
| 50 Flay 2000 (3) | Classroom plus additional component. Tob, Alc, drugs | NR, incl = 1980s and 90s | 18 (13) | Primary and secondary age | None stated. |
| 51 Donaldson 1996 (3) | Tob, Alc, drugs | About 1976-1996 | 78 r | School-age | None stated |
| 52 Montoya 2003 (2) | Tob, Alc, marijuana | About 1980-2003 | 81 r | Adol | None stated |
| 53 Botvin 2000 (2) | Tob, Alc, drugs | About 1980-2000 | 70 r | Primary & secondary age | None stated |
| 54 Paglia 1997 (2) | Tob, Alc, illicit drugs | NR | 176 r | Youth | None stated |
| **Sexuality** | | | | | |
| 55 Robin 2004 (7) | Sex: hiv, std, pregnancy | 1990-1999 | 20 (about 13) | Youth and Adol | RCT or quasi-experimental; control for pretest differences; cell size at least 16; follow-up of at least 4 weeks; attrition less than 40%; behavioral or biological outcome |
| 56 Johnson 2003 (7) | Programs with HIV content. Sex: hiv | Up to 2000 | 44 | Adol 11-18 yrs | RCT or quasi-experimental with rigorous controls; outcome measures relevant to sexual risk behavior; sufficient information to calculate effect sizes. |
| 57 DiCenso 2002 (7) | Sex: pregnancy | 1970-2000 | 26 (13) | Adol 11-18 yrs | RCT; measured initiation of intercourse, use of birth control or pregnancy. Additional quality rating. |
| 58 Mullen 2002 (7) | Sex: hiv | 1992-1998 | 20 (6) | Adol 13-19 yrs | RCT or controlled design with pretest inequivalence controlled; measure behavior or biologic indicator |
| 59 Silva 2002 (7) | Sex | 1985-2000 | 12 (12) | Adol | RCT or quasi-experimental; equivalent no-intervention control group; measure of abstinent behavior; peer-reviewed |
| 60 Yamada 1999 (7) | Sex: std | Up to Sep 1998 (incl= 1992-1998) | 24 (about 10) | 10-19 yrs | RCT or controlled; sample representative of general population; behavioral outcome. Additional quality rating |
| 61 Franklin 1997 (7) | Sex: pregnancy | Up to 1995 | 32 (about 14) | Adol 11-20 yrs | Behavioral measure, peer-reviewed. No design criteria for inclusion. Additional quality rating. |
| 62 Kim 1997 (7) | Sex: hiv | 1983-1995 | 40, 4 in meta (19) | Adol 10-18 yrs | No design criteria for inclusion in review. Additional quality rating. RCT criterion for inclusion in meta-analysis. |
| 63 Oakley 1995 (7) | Sex: sexual health | 1982-1994 | 12 sound-65 | 0-19 yrs | Sound study: RCT or equivalent control group; pre- and post data; reporting of all outcomes |
| 64 Bennett 2005 (6) | Sex: pregnancy | 1980-2002 | 16 (16) | Secondary school | RCT; outcomes sexual behavior, contraceptive knowledge or use, or pregnancy |
| 65 Pedlow 2004 (6) | Sex | Up to Feb 2003 | 24 (10) | 11-18 yrs | RCT; behavioral outcome measure |
| 66 Kirby 2002 (5) | Sex: hiv, std, pregnancy, abstinence | 1980 – 2001 | 73 | 12-18 yrs | (Quasi-)experimental; sample size at least 100 in groups combined; measure of behavior or behavioral outcome. |
| 67 Song 2000 (5) | Sex | 1960-1997 | 67 (67) | Adol Grade 5-12 | Outcome measure of knowledge about sexuality |
| 68 Thomas 2000 (3) | Programs with focus on abstinence. Sex: std, pregnancy | NR, incl = 1990-1995 | 9 (8) | Adol | None stated |
| 69 Milburn 1995 (3) | Peer education programs. Sex. | NR | 51 r | Young people | None stated |
| 70 Christopher 1995 (2) | Sex: pregnancy | NR, incl = 1981-1994 | About 13 | Adol | None stated. |
| 71 Jacobs 1995 (2) | Sex: pregnancy, std | NR, incl = 1982-1991 | 6 (5) | Adol | None stated. |
| **Nutrition** | | | | | |
| 72 Knai 2006 (7) | Nut: fruit, vegetables | Up to Apr 2004 | 15 (4) | Child, Adol 5-18 yrs | Controlled; follow-up at least 3 months; behavioral outcome. Additional quality rating. |
| 73 Shilts 2004 (7) | Programs including goal-setting. Nut, exercise | 1977- Dec 2003 | 28 (1) | All ages; sub sample Adol 12-19 yrs | Experimental, quasi-experimental or pre-experimental (no cross-sectional); cell size greater than 5. Additional quality rating. |
| 74 Thomas 2004 (7) | Nut, exercise. Subset of dietary programs. | 1985-Aug 2003 | 57 (4) | 6-18 yrs | Prospective controlled studies; behavioral outcome. Additional quality rating. |
| 75 Ammerman 2002 (7) | Nut: fat, fruit, vegetables | 1975-Aug 1999 | 92 (1) | All ages; sub sample school-based | RCT or quasi-experimental; behavioral outcome; sample size at least 40; diet freely chosen by participants. Additional quality rating. |
| 76 Ciliska 1999 (7) | Nut: fruit, vegetables | Up to Aug 1998 | 15 (1) | > 4 yrs | Prospective study with comparison group; information on process or outcome evaluation. Additional quality rating. |
| 77 Contento 1995 (7) | Nut | 1980-1995 | 43 (about 20) | School-age | Random or quasi-experimental design; evidence of instrument reliability and validity |
| 78 French 2003 (6) | Environmental programs. Nut: fruit, vegetables | NR, incl = 1993-2003 | 11 (2) | Primary and secondary age | No criteria stated. All included studies are controlled, about half randomized |
| 79 Hoelscher 2002 (3) | Nut | 1994-2000 | 17 (about 5) | Adol 11-18 yrs | No criteria stated |
| 80 Lytle 1995 (3) | Nut | 1980-1995? | 85 r | Child, Adol | Controlled; behavioral outcome; peer-reviewed |

*Note. Within behavior categories, reviews are ordered by quality rating and publication year.*

*^a^ Reference number, first author, publication year, quality score in parentheses (0-3=weak, 4-5=moderate, 6-7=strong). ^b^ Behavior focus: Alc=alcohol, Nut=nutrition, Sex=sexuality, Tob=tobacco, std=sexually transmitted disease. If applicable, a more specific focus is recorded for sexuality and nutrition reviews (e.g., pregnancy, fat consumption). ^c^ Time span used in search strategy. NR=not reported, Incl=actual time span of included studies, recorded for reviews that did not report time span of search strategy. ^d^ For reviews that did not report the number of included primary studies the total number of references is given, indicated by r. Numbers between parentheses indicate the number of primary studies meeting relevance criteria of the present review (target behaviors, secondary school-age, school-based educational intervention). This number was only examined for reviews that provided sufficient information about primary studies. ^e^ Child=children, Adol=adolescents, yrs=years. ^f^ RCT=randomized controlled trial*

**Table S2. Elements of program focus related to program effectiveness: results of reviews by d**omain

| **Element** | **Multiple domains** | **Substance abuse** | **Sexuality** | **Nutrition** |
| --- | --- | --- | --- | --- |
| Focus on specific behavior |  | 52 (2): +. | 56 (7): +. 66 (5): +. | 72 (7): +. 74 (7): +. 76 (7): +. 77 (7): +. 79 (3): +. |
| Abstinence-plus (AP) vs abstinence-only (AO) |  | 35 (7): +/?. 54 (2): +. | 59 (7): AP vs AO 0. 61 (7): AP +. 63 (7): AO -. 64 (6): AP vs AO ?. 66 (5): AO ?. 68 (3): AO 0, AP +. 70 (2): AO 0. |  |

*Note. Reviews are indicated by the reference number and, between parentheses, the quality rating (0-3=weak, 4-5=moderate, 6-7=strong). Results are indicated by the following characters: +=positive, -=negative, 0=null, ?=unclear contribution to program effectiveness.*

***Table S3. Elements of program development related to program effectiveness: results of reviews by domain***

| **Element^a^** | **Multiple domains** | **Substance abuse** | **Sexuality** | **Nutrition** |
| --- | --- | --- | --- | --- |
| **Use of theory** | 26 (7): ?. 29 (2): +. | 36 (7): +. 38 (7): +. 40 (7): +. 42 (7): 0. 47 (5): +. 53 (2): +. | 56 (7): +/0. 60 (7): +. 62 (7): +. 66 (5): +. 68 (3): +. 70 (2): +. | 76 (7): +. 77 (7): +. 79 (3): +. 80 (3): +. |
| Social cognitive theory | 29 (2): +. | 36 (7): +. 40 (7): +. 47 (5): +. 53 (2): +. | 70 (2): +/?. | 77 (7): +. 79 (3): +. 80 (3): +. |
| Addressing determinants | 29 (2): +. | 52 (2): +. 53 (2): +. 54 (2): +. | 66 (5): +. | 77 (7): +. |
| Conducting needs assessment | 26 (7): + | 35 (7): +. |  |  |
| Students involved in planning, implementation | 26 (7): +. | 54 (2): +. |  |  |
| Formative phase, interviews, pretesting |  | 35 (7): +. | 56 (7): 0. |  |
| Tailoring to culture, ethnicity |  | 31 (7): ?. 33 (7): +. 41 (7): +. 47 (5): +. 53 (2): +. | 66 (5): +. 29 (2): +. | 79 (3): ?. |
| Tailoring to cognitive ability or age |  | 31 (7): ?. | 65 (6): +. | 77 (7): +. 79 (3): +. |

*Note. Reviews are indicated by the reference number and, between parentheses, the quality rating (0-3=weak, 4-5=moderate, 6-7=strong). Results are indicated by the following characters: +=positive, -=negative, 0=null, ?=unclear contribution to program effectiveness.*

*^a^ Elements marked bold are considered by us to have similar results in the domains of substance abuse, sexuality and nutrition.*

***Table S4. Elements of program content related to program effectiveness: results of reviews by domain***

| **Element^a^** | **Multiple domains** | **Substance abuse** | **Sexuality** | **Nutrition** |
| --- | --- | --- | --- | --- |
| Knowledge, risk, attitudes | | | | |
| Knowledge only approach | 26 (7): 0. 29 (2): 0. | 31 (7): 0. 40 (7): 0. 41 (7): 0. 47 (5): 0. 50 (3): 0. 51 (3): 0. 53 (2): 0. 54 (2): 0. | 29 (2): 0. | 74 (7): 0. 76 (7): 0. 77 (7): 0. |
| Factual information |  | 54 (2): +. | 60 (7): +. 66 (5): +. 70 (2): +. |  |
| Short-term consequences | 29 (2): +. | 54 (2): +. |  |  |
| Enhancing perceived risk |  | Fear arousal: 47 (5): 0. 51 (3): 0. 54 (2): 0. | 58 (7): 0. 65 (6): +. |  |
| Social influences | | | | |
| **Social influences** | 29 (2): +. | 31 (7): 0/+. 32 (7): +. 35 (7): +. 39 (7): + especially for girls. 40 (7): +. 41 (7): +. 43 (7): +. 50 (3): +. 51 (3): +. 52 (2): +. 53 (2): +. | 65 (6): +. 66 (5): +. 68 (3): +. | 77 (7): +. 79 (3): + with younger adolescents. |
| **Social norms** | 29 (2): +. | 35 (7): +. 36 (7): +. 50 (3): +. 51 (3): +. 52 (2): +. 54 (2): +. | 65 (6): +. 68 (3): +. | 77 (7): +. |
| Resistance skills | 26 (7): +. 28 (6): behavior specific +, general 0. 29 (2): +. | 35 (7): ?. 36 (7): 0. 38 (7): not suited for alternative high school students. 28 (6): for tob + when embedded, for alc 0/+ when embedded. 47 (5): +. 51 (3): 0/+. 50 (3): 0/+. 52 (2): +.  54 (2): 0. | 28 (6): 0/-. 66 (5): +. 68 (3): +. |  |
| Skills | | | | |
| Skills (unspecified) |  |  | 61 (7): ?. | 77 (7): +. 79 (3): +. 80 (2): +. |
| Practical domain-bound skills (condom use, food preparation) |  |  | 55 (7): +. 56 (7): +. 65 (6): ?. | 72 (7): + food preparation skills. |
| **Cognitive-behavioral program / skills training** |  | 32 (7): +. 38 (7): TND program +. 53 (2): +. | 65 (6): +. | 77 (7): +. 79 (3): +. |
| Life skills (self-management, decision-making, social and assertive skills, anxiety manag) | 29 (2): +. | 31 (7): 0/+. 32 (7): +. 33 (7): 0/+. 36 (7): +. 40 (7): +. 41 (7): +. 47 (5): +. 51 (3): +. 52 (2): +. 53 (2): +, without drug focus 0. 54 (2): +. |  |  |

*Note. Reviews are indicated by the reference number and, between parentheses, the quality rating (0-3=weak, 4-5=moderate, 6-7=strong). Results are indicated by the following characters: +=positive, -=negative, 0=null, ?=unclear contribution to program effectiveness.*

*^a^ Elements marked bold are considered by us to have similar results in the domains of substance abuse, sexuality and nutrition.*

**Table S5. Elements of program methods related to program effectiveness: results of reviews by d**omain

| **Element** | **Multiple domains** | **Substance abuse** | **Sexuality** | **Nutrition** |
| --- | --- | --- | --- | --- |
| Multiple channels or strategies | 29 (2): +. |  | 65 (6): +. 66 (5): +. |  |
| Didactic style (lecture) |  | 54 (2): 0. |  | 72 (7): 0. |
| Interactive (incl. discussion, role play) | 28 (6): + discussion. 29 (2): + discussion, role play. | 35 (7): +. 36 (7): +. 40 (7): +. 41 (7): +. 51 (3): +. 54 (2): +. | 68 (3): + role play. | 79 (3): + discussion. |
| (Cognitive)behavioral skills training |  | 53 (2): +. | 65 (6): +. |  |
| Active, experiential learning |  | 54 (2): +. |  | 72 (7): +. 77 (7): +. 80 (3): +. |
| Having students personalize info (e.g., diet self-assessment) |  |  | 66 (5): +. | 77 (7): +. 79 (3): +. 80 (3): +. |

*Note. Reviews are indicated by the reference number and, between parentheses, the quality rating (0-3=weak, 4-5=moderate, 6-7=strong). Results are indicated by the following characters: +=positive, -=negative, 0=null, ?=unclear contribution to program effectiveness.*

***Table S6. Elements of program facilitator related to program effectiveness: results of reviews by domain***

| **Element^a^** | **Multiple domains** | **Substance abuse** | **Sexuality** | **Nutrition** |
| --- | --- | --- | --- | --- |
| Peer leader | 26 (7): +/?. 28 (6): +. 29 (2): +. | 34 (7): +/0. 35 (7): 0. 36 (7): +/0. 40 (7): +/0. 41 (7): 0. 43 (7): +. 45: (6) +/0. 47 (5): +. | 56 (7): 0. 59 (7): 0. 68 (3): +. 69 (3): ?. | 72 (7): +. |
| Teacher | 28 (6): +. | 34 (7): 0. 35 (7): 0. 41 (7): 0. 47 (5): +. | 59 (7): 0. |  |
| Peer vs teacher same program | 26 (7): +/0. | 31 (7): ?. 37 (7): +/0. | 55 (7): 0/?. |  |
| **Trained facilitator** |  | 35 (7): +. 47 (5): +. | 55 (7): +. 60 (7): +. 66 (5): +. | 72 (7): +. 77 (7): +. |

*Note. Reviews are indicated by the reference number and, between parentheses, the quality rating (0-3=weak, 4-5=moderate, 6-7=strong). Results are indicated by the following characters: +=positive, -=negative, 0=null, ?=unclear contribution to program effectiveness.*

*^a^ Elements marked bold are considered by us to have similar results in the domains of substance abuse, sexuality and nutrition.*

**Table S7. Elements of program components related to program effectiveness: results of reviews by d**omain

| **Element^a^** | **Multiple domains** | **Substance abuse** | **Sexuality** | **Nutrition** |
| --- | --- | --- | --- | --- |
| **Multi-component programs** | 27 (7 S): +. 29 (2 S): +. | 31 (7 S): 0/+. 32 (7 S): ?. 36 (7 S): +. 40 (7 S): +. 50 (3 S): 0/?. 51 (3): +. 54 (2): +. | 56 (7): +. 57 (7): 0. 59 (7 S): +. 61 (7): +. 65 (6): +. 66 (5): +. 68 (3): +. 70 (2): +/?. 71 (2 S): +. | 72 (7): +. 74 (7): +. 76 (7): +. 77 (7): +. 78 (6 S): +/0. 79 (3): +. 80 (3): +. |
| School wide change and family or community component |  | 40 (7 S): +. |  | 77 (7): +. 78 (6 S): +/0. 79 (3): +. 80 (3): +. |
| Community interventions |  | 33 (7): +/?. 54 (2): +. | 61 (7): +. 66 (5): +. | 80 (3): +. |
| Community component additional to school | 29 (2 S): +. | 31 (7 S): 0/+. 32 (7 S): ?. 36 (7 S): +. 50 (3 S): 0/?. 51 (3): +. 54 (2): +. |  | 72 (7): +. 77 (7): +. |
| School-wide activities |  | 50 (3 S): 0/?. 52 (2): +. 54 (2): ?. | 70 (2): +/0. 71 (2 S): +. | 72 (7): +. 74 (7): +. 78 (6 S): +/0. 79 (3): +. 80 (3): +. |
| Parent / family involvement |  | 31 (7 S): 0/+. 33 (7): high-risk +. 36 (7 S): +. 40 (7 S): +. 50 (3 S): general population 0/?, high-risk +. 52 (2): +. 54 (2): general population 0, high-risk 0/+. | 59 (7 S): +. 65 (6): +. 68 (3): +. | 72 (7): +. 76 (7): +. 74 (7): +. 77 (7): elementary school +, middle/high school 0. 78 (6 S): +/0. 79 (3): +. 80 (3): elementary age +. |
| Policy: price regulation |  | 52 (2): +. 54 (2): +. |  | 78 (6 S): +. |

*Note. Reviews are indicated by the reference number and, between parentheses, the quality rating (0-3=weak, 4-5=moderate, 6-7=strong); reviews with a specific focus on schools are indicated by S between parentheses. Results are indicated by the following characters: +=positive, -=negative, 0=null, ?=unclear contribution to program effectiveness.*

*^a^ Elements marked bold are considered by us to have similar results in the domains of substance abuse, sexuality and nutrition.*

**Table S8. Elements of program intensity related to program effectiveness: results of reviews by d**omain

| **Element** | **Multiple domains** | **Substance abuse** | **Sexuality** | **Nutrition** |
| --- | --- | --- | --- | --- |
| Intensity |  | 31 (7): ?. 36 (7): ?. | 55 (7): +/?. |  |
| Duration |  | 34 (7): 0. 54 (2): ?/+. | 55 (7): +/?. 66 (5): +. | 72 (7): + (at least 12 months). |
| Number of sessions or hours |  | 35 (7): ?. 38 (7): TND 9 sessions 0 for tob and + for alc (only baseline nonusers alc); 12 sessions + for tob and alc (only baseline nonusers alc). 40 (7): +/0. 41 (7): 0. 47 (5): LST 15 sessions +. 48 (5): 10 sessions in first yr and 5 sessions in second yr +. | 58 (7): 0. 59 (7): 0. 60 (7): minimum of 8 hours +. 62 (7): +. 55 (7): +/?. | 74 (7): + (at least 10 sessions). 76 (7): +. 77 (7): + (10-15 hrs is too little). 79 (3): +. 80 (3): +. |
| Boosters, continued education | 29 (2): +. | 35 (7): +. 36 (7): ?. 43 (7): ?. 44 (6): +/0. 47 (5): 15 booster sessions +. 48 (5): +. 51 (3): +. 53 (2): +. 54 (2): +. | 65 (6): +. |  |

*Note. Reviews are indicated by the reference number and, between parentheses, the quality rating (0-3=weak, 4-5=moderate, 6-7=strong). Results are indicated by the following characters: +=positive, -=negative, 0=null, ?=unclear contribution to program effectiveness.*
